# The Value of Real-World Data in Understanding Prostate Cancer Risk and Improving Clinical Care: Examples from Swedish Registries

**DOI:** 10.3390/cancers13040875

**Published:** 2021-02-19

**Authors:** Kerri Beckmann, Hans Garmo, Ingela Franck Lissbrant, Pär Stattin

**Affiliations:** 1 Cancer Epidemiology and Population Health Research Group, Allied Health and Human Performance, University of South Australia, Adelaide, SA 5001, Australia; kerri.beckmann@unisa.edu.au or; 2 Translational Oncology and Urology Research, School of Cancer and Pharmaceutical Studies, Kings College London, London SE1 9RT, UK; hans.garmo@kcl.ac.uk; 3 Regional Cancer Centre, Uppsala University Hospital, SE-75122 Uppsala, Sweden; 4 Department of Oncology, Institute of Clinical Sciences, Sahlgrenska University, SE-41345 Gothenburg, Sweden; ingela.franck.lissbrant@oncology.gu.se; 5 Department of Surgical Sciences, Uppsala Hospital University, SE-75185 Uppsala, Sweden

**Keywords:** prostate cancer, management, risk, real-world data, clinical registry, administrative data

## Abstract

**Simple Summary:**

Real-world data (RWD), i.e., data reflecting normal clinical practice collected outside the constraints of randomised controlled trials, provide important insights into our understanding of prostate cancer and its management. Clinical cancer registries are an important source of RWD. Depending on their scope and the potential linkage to other data sources, registry-based data can be utilised to address a variety of questions including risk factors, healthcare utilisation, treatment effectiveness, adverse effects, disparities in healthcare access, quality of care and healthcare economics. This review describes the various registry-based RWD sources for prostate cancer research in Sweden (namely the National Prostate Cancer Register, the Prostate Cancer data Base Sweden (PCBaSe) and the Patient-overview Prostate Cancer) and documents their utility for better understanding prostate cancer aetiology and improving clinical care.

**Abstract:**

Real-world data (RWD), that is, data from sources other than controlled clinical trials, play an increasingly important role in medical research. The development of quality clinical registers, increasing access to administrative data sources, growing computing power and data linkage capacities have contributed to greater availability of RWD. Evidence derived from RWD increases our understanding of prostate cancer (PCa) aetiology, natural history and effective management. While randomised controlled trials offer the best level of evidence for establishing the efficacy of medical interventions and making causal inferences, studies using RWD offer complementary evidence about the effectiveness, long-term outcomes and safety of interventions in real-world settings. RWD provide the only means of addressing questions about risk factors and exposures that cannot be “controlled”, or when assessing rare outcomes. This review provides examples of the value of RWD for generating evidence about PCa, focusing on studies using data from a quality clinical register, namely the National Prostate Cancer Register (NPCR) Sweden, with longitudinal data on advanced PCa in Patient-overview Prostate Cancer (PPC) and data linkages to other sources in Prostate Cancer data Base Sweden (PCBaSe).

## 1. Introduction

Prostate cancer (PCa) poses many research challenges for understanding aetiology, natural history and the most effective treatments since clinical practice has changed dramatically over the past two decades. Evidence to improve our understanding and control of the most common non-skin cancer affecting males has been generated through a variety of approaches including biomedical research, clinical trials and observational (non-experimental) studies using different methodologies and data sources.

Randomised controlled trials (RCTs) constitute the highest level of evidence to inform guideline development for early detection, diagnosis and management of PCa [1]. RCTs are the best study design for establishing causal inferences and testing the efficacy of interventions in clinical care [2]. However, not all research questions regarding PCa can be addressed through RCTs. For example, researchers cannot intervene to determine the natural history of a disease, identify “harmful” risk factors, assess long-term outcomes and rare adverse events, determine trends in management, identify disparities in access to treatments or examine guideline compliance. These are aspects of knowledge about PCa that can only be addressed using real-world data (RWD) [3].

The objective of this review is to demonstrate the value of RWD to advance knowledge of PCa aetiology, patterns of care and treatment effectiveness and to inform clinical practice to improve outcomes. This review focuses primarily on RWD from registries in Sweden. Following a general overview of the value and limitations of RWD, the use of RWD to better understand PCa and improve care is illustrated by drawing on examples of studies using data from Swedish registries. Finally, this review describes future directions for enhancing the capacity of these registries to provide real-world evidence for PCa research.

## 2. What Is Real World Data (RWD) and What Contribution Does It Make?

RWD is defined by the Association of the British Pharmaceutical Industry as “data that are collected outside the controlled constraints of conventional RCTs to evaluate what is happening in normal clinical practice” [4]. This includes data from patient or disease registers, observational studies including prospective and retrospective study designs, electronic health records, administrative datasets (e.g., claims data) and patient-reported outcome surveys (PROs), along with data from pragmatic trials (i.e., clinical trials to test effectiveness within the broader community).

Both RWD and RCTs have specific strengths and limitations. RCTs primarily focus on maximising internal validity in order to establish causal relationships. Randomisation is key to limiting indication bias by ensuring even distribution of known and unknown confounding factors between comparison groups. Internal validity is also maximised through clear protocols for care in both intervention and comparison arms and through selection of a homogenous study population with high likelihood of compliance [5].

However, findings from RCTs conducted under ideal conditions with selective patient populations limit the generalisability to the broader population in clinical practice [5]. RCTs are expensive, sometimes struggle to recruit participants and frequently are not completed [6,7]. Even when completed, the lengthy processes involved may result in RCT findings that are no longer relevant to current practice. Due to time and cost constraints, RCTs are often not sufficiently powered or have too short a follow-up to assess adverse effects of specific interventions. Furthermore, some healthcare interventions are widely applied in clinical practice without RCT evidence, making it difficult to establish “ideal” trial conditions. Such interventions are never likely to be evaluated in RCTs.

In contrast to RCTs, studies utilising RWD generally costs less, can address a broader range of questions, provide more timely evidence and include a broader cross-section of the population; hence, they are more generalisable to the community [8]. In addition, they provide the only means of studying risk factors/exposures that cannot be controlled (natural history/aetiology/biomarkers). Given the potential for longer follow-up and larger numbers to identify rare outcomes, RWD provide an efficient way to undertake post-approval surveillance studies of medical interventions [9]. RWD also enable assessment of the comparative effectiveness of well-established treatments where little RCT data exist. Such studies are important in PCa given the variety treatment options and sparsity of head-to-head trials comparing modalities. RWD are essential for studying healthcare utilisation and costs as well as social determinants and disparities in access, quality of care and outcomes, i.e., questions that cannot be addressed through RCTs.

Despite these opportunities, RWD have some limitations. Since RWD reflect real-world practice, they include inherent biases in patient selection for specific treatments. While advanced analytical methods (e.g., multivariable methods, propensity matching, marginal structural modelling) and increased computing capacities help limit confounding/selection bias, it is not always possible to remove these biases completely [3]. Hence, treatment efficacy is better established through RCTs. However, RWD play a crucial role in establishing treatment effectiveness, particularly since key subpopulations for whom these treatments are intended are often excluded or underrepresented in RCTs. Such studies are of great importance since not all efficacious cancer control measures are effective within the broader population who ultimately receive them.

The scope and quality of evidence generated using RWD are limited by the availability and quality of data within respective datasets. Many RWD sources are secondary sources, meaning that data are collected for purposes other than research, such as reimbursement. Limitations include missing relevant data, inaccurate or incomplete coding and changes to coding systems over time [10,11]. RWD can also lack representativeness if they apply to restricted subgroups of the population (e.g., USA Medicare data) [12]. Even with population-wide data sources, findings in one jurisdiction may not be generalisable to another due to differences between healthcare systems, policies and practices across regions. Hence, careful consideration needs to be given to the quality, coverage and representativeness of RWD sources.

Cancer registries collect RWD within a defined population. They can be disease-, health service- or product-specific [12]. Given the level of detail collected, disease-specific clinical registries tend to support a variety of research questions, including incidence and mortality trends, disease characteristics, risk factors, healthcare utilisation, disparities in access to services, quality of care, health-related quality of life and health economics [13].

The Surveillance, Epidemiology and End Results (SEER) register, established in 1971 by the National Cancer Institute, USA, is a well-known example of a population-based register providing RWD to address clinical questions in cancer care [14]. SEER comprises data from selected state- or regional population-based cancer registries across the United States. It includes information on stage, grade and initial treatment across all cancer types. SEER (2013) data pertain to about 30% of the US [14]. Over 1,000,000 men with PCa are included; however, lacking or incomplete data on risk categories, comorbidities, subsequent treatments, prostate-specific antigen (PSA) levels and follow-up limit SEER’s usefulness for monitoring disease progression and mapping the disease trajectory [12].

Data from the SEER registry have been greatly enhanced through data linkage with other administrative databases. For example, SEER-Medicare has linked SEER registries with claims data from Medicare (the national health insurance programme for US residents) to provide greater clinical detail and potential for follow-up of treatment trajectories [15]. SEER-Medicare includes information on hospital admissions, drug prescriptions, comorbidities, subsequent treatments and healthcare utilisation and costs, both before and after cancer diagnosis.

Additional refinements have been made to SEER databases. SEER now also links to other datasets which provide survey responses and, thus, provide important information on the patient experience [16]. Recent updates of SEER-Medicare now include a 5% sample of cancer-free controls [17]. The value these RWD sources in understanding and improving cancer care is considerable, with the number of scientific publications on cancer care using SEER or SEER-Medicare data now exceeding 1800 [17].

The remainder of the review showcases similarly valuable, registry-based RWD sources from Sweden.

## 3. Real-World Prostate Cancer Data from Sweden

Several “real world” population-based data on PCa are available in Sweden, namely within the National Prostate Cancer Register (NPCR) and through Prostate Cancer data Base Sweden (PCBaSe), which is based on linkages between NPCR and numerous other national registers in Sweden. In addition, longitudinal follow-up of men with advanced disease is now also accessible through the recent establishment of the Patient-Overview Prostate Cancer (PPC) sub-register. Interested researchers can apply to access these data via the NPCR (npcr@npcr.se).

### 3.1. National Prostate Cancer Register (NPCR) of Sweden

The NPCR has recorded detailed disease-specific data on clinical characteristics, treatments and outcomes for men diagnosed with PCa across Sweden since 1998 [18,19]. Quality assessments of the NPCR indicate very high coverage (98% compared with the Swedish Cancer Register, which has mandated reporting) and completeness (90% across 48 data items) [20,21]. This population-based register focuses on clinical characteristics, diagnostic pathways and primary treatment within the first six months of diagnosis. Major limitations include the lack follow-up data on clinical and biochemical recurrence and subsequent treatments. Since 2008, the NPCR has collected detailed information on radical radiotherapy (primary, secondary and post-operative treatment), with data before that period being collected through a retrospective audit of radiotherapy services across Sweden, RETRORAD [22]. Since 2015, comprehensive information on primary and secondary radical prostatectomy (RP) has also been collected in the NPCR, including pathological assessment after RP [23].

### 3.2. Prostate Cancer Data Base Sweden (PCBaSe)

The utility of the NPCR as a source of RWD to drive clinical research has been greatly enhanced through the creation of Prostate Cancer data Base Sweden (PCBaSe) [19]. This database links NPCR with other national registers in Sweden, using individuals’ unique personal identification number. Core national registers within PCBaSe include the National Patient Registry (NPR), available from 1987, the national Prescribed Drug Registry (PDR), available from July 2005, the Longitudinal Integration Database for Insurance and Labour Market Studies (LISA) from 1990 and the Multi-Generation Registry (i.e., family membership for individuals born after 1932), the National Cause of Death Registry from 1953 and the National Cancer Registry from 1958. These linkages provide access to data on a far greater range of factors—for example, comorbid conditions, additional treatments and serious adverse effects via hospital admission records, prescribed medications, including hormonal therapies but also other medications, family history of PCa registry, employment and civil status and other cancer diagnoses. An advantage of PCBaSe is the national coverage of most other “secondary” data sources such as the Prescribed Drug Register and the Patient Register due to Sweden having a national, tax-financed healthcare system, thus minimising the potential for bias due to the inclusion/exclusion of selective populations. This is not always the case in other linked registry systems—for example, SEER-Medicare, where restrictions apply with regard to eligibility for Medicare (>65 years or disabled) [17]. In addition to PCa cases, PCBaSe (v2.0 onwards) has included five PCa-free men, matched by year of birth and region of residence with respective cases, to serve as a comparison population. Comparison men are randomly selected within matching strata from the Population Register by Statistics Sweden. Two sets of PCa-free men are selected: one set with replacement to facilitate case–control studies and one set without replacement for cohort studies.

While not a prospective data repository, both historical and longitudinal data for cases and comparison men can be assembled to provide a more comprehensive picture of treatment patterns. This is exemplified through PCBaSe^Traject^ [18]. PCBaSe^Traject^ provides detail on the treatment trajectory for men with PCa who were alive in January 2006, (~100,000 PCa cases), including primary and subsequent treatments during follow-up. However, PCBaSe^Traject^ does not include data on indications for additional treatments, PSA follow-up and in-hospital chemotherapies [18].

**Figure 1:**
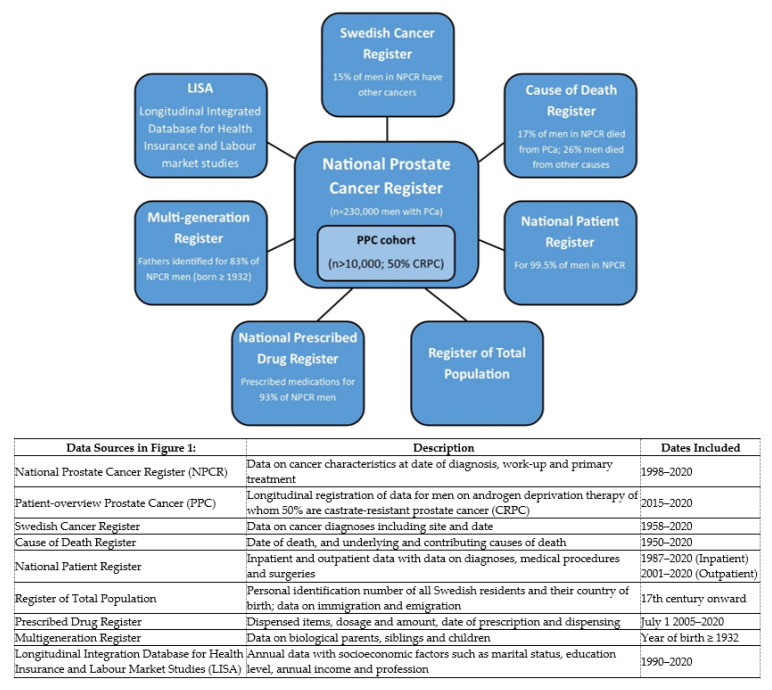
National healthcare and demographic registers to be included in Prostate Cancer data Base Sweden (PCBaSe) 5.0 in 2021.

The PCBaSe platform is updated at three-year intervals. Figure 1 outlines the datasets for inclusion in the next release (PCBaSe_5.0), due in mid-2021. This version will include all men diagnosed in the period 1998–2020 (~230,000 men) plus selected controls, with follow-up to December 31 2020. “Rapid” linkages are performed annually to include men diagnosed during the previous year (e.g., PCBaSe RAPID-2019, created in June 2020, included men diagnosed with PCa up to December 31 2019) to allow assessment of trends in treatments.

### 3.3. Patient-Overview Prostate Cancer (PPC)

In 2015, the NPCR team established a sub-register of patients with advanced PCa, known as the Patient-overview Prostate Cancer (PPC) [24]. This “voluntary” register incorporates longitudinal follow-up of men with advanced PCa, including those with castrate-resistant prostate cancer (CRPC), until death. The current number enrolled in PPC exceeds 10,000. Though not population-based, characteristics of this cohort have been shown to be representative of men within the NPCR who receive androgen receptor-targeted drugs (ARTs) [24].

**Figure 2:**
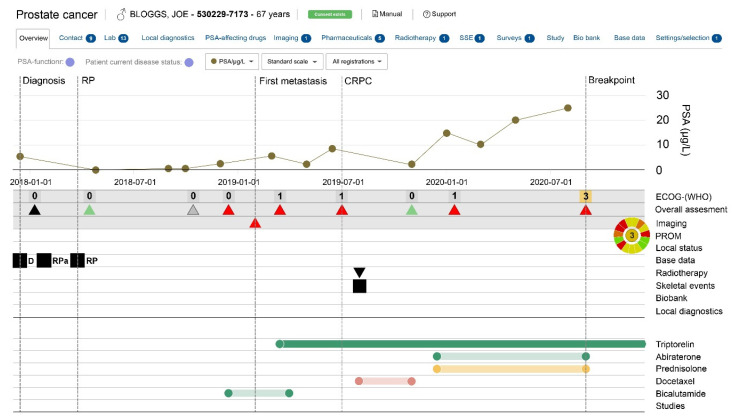
Example of the clinical “decision aid” generated for a fictitious patient in the Patient-overview Prostate Cancer (PPC). Legend: Red = poor performance; yellow acceptable performance/ green = good performance; Full colour shows ongoing drug, weak colour shows previous drugs use that has stopped; D = Date for diagnosis; RPa = Date of decision to perform radical prostatectomy; RP = Date radical prostatectomy performed.

Updates of patient status and treatments are provided by treating clinicians/staff at each patient visit. Data items collected include date of castrate resistance, start and stop dates for all PCa treatments and results of imaging and laboratory tests (e.g., PSA kinetics, skeletal events and assessment of Eastern Cooperative Oncology Group (ECOG) performance status [25]). Since 2018, patient-reported outcome measures (PROMs) have been collected at each clinical appointment, using a modified version of the EORTC-C15-PALD-5L [26], to assess physical functioning, symptoms and wellbeing. As a subset of the NPCR, PPC is also part of PCBaSe and, as such, includes data on comorbidities, adverse events, healthcare utilisation, precise dates, dosage and quantity of filled medications (from PDR) impact on work status, sick leave, etc. (from LISA), and cause of death through the Cause of Death Register. Importantly, PPC provides data on drugs delivered in-hospital that are not included in the PDR, and PROMs can be assessed in relation to changing therapies. In addition, PPC assists clinicians to see a complete treatment history by providing them with a graphical summary of treatments, procedures and outcomes for any patients they have enrolled in the register. Figure 2 show an example of the “PPC decision aid” for an individual man.

## 4. The Value of RWD in Understanding PCa—Examples from Swedish Registers

### 4.1. Prostate Cancer Aetiology

One important area of research where data from non-trial settings are essential is understanding of the aetiology of PCa. Very few risk factors for PCa have been identified, the exceptions being race and familial risk. Identifying risk factors and understanding the level of risk is only possible through exploration of RWD sources since exposures cannot be randomly assigned as in RCTs. The same applies to investigating risk factors that are not modifiable or considered hazardous (e.g., fatherhood, height, smoking, obesity and other comorbid conditions).

The inclusion of PCa-free men within PCBaSe [19] has spawned numerous case–control studies investigating risk factors and protective factors for PCa. The scope of such studies is extremely broad owing to the range of datasets currently included. To date, most studies have investigated risks associated with other comorbid diseases (e.g., diabetes [27]) or specific medications (e.g., 5-apha reductase inhibitors for benign hyperplasia [28], testosterone replacement therapy [29], anti-inflammatory medications [30], antidiabetic medications [31] and the antihypertensive drug spironolactone, known to have antiandrogenic effects [32]). In addition, case–control studies using PCBaSe have also examined immigrant status [33], fatherhood status [34,35] and family history of PCa [36].

Several studies investigating familial risk have utilised the linkages in PCBaSe between the NPCR and the Multi-Generation Register, which identifies family links across generations of men [36,37,38]. In their 2012 paper, Jansson et al. established that brothers of men with high-grade PCa were at greater risk of being diagnosed with high-grade PCa as compared to the general population [38]. Their follow-up study examined the level of concordance for high-risk disease according to sibling relationships, with monozygotic twins having the highest risk and maternal half-brothers having the lowest risk [37]. Bratt et al. found that increased diagnostic activity in family members also contributed to the familial aggregation of prostate cancer [39]. Men who had a brother with PCa were at particularly high risk of being diagnosed with low-risk PCa in the first year after the date of his brother’s diagnosis. A subsequent study by the same team provided estimates of the age-specific probability of intermediate–high-risk PCa according to brothers’/fathers’ PCa status [36]. These data are important when counselling men about their familial risk.

The ease of linking data across different national registers using unique identity numbers has provided further scope to investigate the interplay between PCa and other diseases utilising RWD. For example, by linking the NPCR with the Kidney Transplant Register, researchers established that there was no additional risk of PCa in transplant recipients who were on immunosuppressive therapy, nor any negative impact on survival in men who were diagnosed with PCa [40].

The potential to also link the NPCR/PCBaSe platform with previous cohort studies has enabled the study of lifestyle factors/health behaviours (e.g., physical activity, diet, body weight and sleep) which are not often collected by registries. One example is the study by Wilson et al., linking NPCR to the Swedish Construction Worker Cohort, which collected health behaviour data at workers’ health checks [41]. This study examined the impact of smoking and use of “snus” (smokeless tobacco) on risk of PCa mortality and found both to be associated with increased risk of PCa death. A similar approach was used by Jochems et al. to study PCa risk and mortality associated with men’s height and body mass index (BMI) using data from five existing study cohorts linked to the NPCR [42].

As a high-quality disease-specific register [20,21], the NPCR has near-complete detailed data for the population on diagnostic characteristics, including clinical stage and PSA levels for newly diagnosed PCa patients, which is often limited in other large/national cancer incidence registers, e.g., SEER registries [43]. This allows for classification by risk category and mode of presentation. These are important distinctions in PCa research, due to the high prevalence of PSA testing leading to detection of low-risk disease. In the study by Jochems et al. [42] on risk associated with height and BMI, researchers were able to stratify by risk category and mode of detection to further refine their analyses. Findings from the stratified analyses suggest that the increased risk of PCa overall among taller men may be due to higher uptake of PSA testing (increased low-risk disease), while the inverse association with BMI may be due to lower uptake of PSA testing (i.e., detection bias). Other PCBaSe studies on risks associated with various medications (e.g., testosterone replacement therapy [29]; anticoagulants [44]) also suggest that detection bias often explains associations, based on analysis of detailed diagnostic information.

### 4.2. Outcomes/Comparative Effectiveness

As a quality registry with population-wide coverage and extensive clinical data, the NPCR is well placed to monitor and compare PCa treatment outcomes. One of the earliest outcome studies in the NPCR assessed population-wide outcomes for men with localised PCa [45] according to treatment approach. This study reported 10-year cumulative mortality among men with low–intermediate-risk PCa who recieved treatment (radical prostatectomy (RP) or radiotherapy) and men who underwent surveillance. While mortality was marginally higher for men who were not treated compared to those who received radical treatment, their mortality did not differ from men in the general population. This report provided support for active surveillance (AS) at a time when there was less widespread acceptance of it as an appropriate management option. Likewise, findings reported by Akre et al. [46] showing high rates of PCa mortality among non-curatively treated men with locally advanced PCa have resulted in increased use of radical radiotherapy instead of hormone therapy only for advanced PCa [47]. Further studies (including RCTs, e.g., Swedish Prostate Cancer Group (SPCG)-15 [48]) to evaluate the benefit of radiotherapy versus prostatectomy for locally advanced PCa are underway.

Several studies have directly compared curative treatment options for localised PCa (RP versus radiotherapy) in terms of mortality [49] and long-term adverse effects [50]. Utilising the rich clinical data collection in PCBaSe to adjust for sociodemographic factors, clinical characteristics and comorbidity, Robinson et al. [49] found no statistically significant differences in PCa mortality between treatment approaches for men with high-risk disease and non-significant findings favouring RP for men with low to intermediate-risk PCa. The ability to adjust for confounding factors afforded through PCBaSe may explain differences with other studies in which outcomes consistently favour prostatectomy [51]. Similarly, Fridriksson et al. [50] used linkages within PCBaSe to assess long-term adverse outcomes requiring hospital admissions/procedures following radiotherapy or RP. Their findings indicated severe adverse effects associated with both treatment modalities, which remained 12 years after treatment, with urinary incontinence being a long-term problem in men who underwent RP, while lower urinary tract obstruction and gastrointestinal problems persisted for men who underwent radiotherapy [50].

Recently, Pettersson et al. [52] compared mortality outcomes across different radiotherapy delivery modalities for localised PCa utilising RETRORAD, an audit of radiotherapy data across Sweden [22]. They found favourable outcomes for high-dose brachytherapy plus external beam radiotherapy (EBRT) compared with conventional fractionated EBRT (70–78 Gy) but did not confirm recent RCT results indicating improved survival with moderately dose-escalated EBRT [53].

### 4.3. Adverse Events/Post-Approval Surveillance

Recently, there has been a rapid increase in the approval of new systemic therapies by the US Food and Drug Administration (FDA) and European Medical Agency (EMA), including many specific systemic therapies to target metastatic PCa [54]. While RCTs provide the strongest evidence for the efficacy of new medications and procedures, which is required for approval/release, they do not lend themselves to a full assessment of safety aspects due their relatively short follow-up times, restricted eligibility criteria and often delayed onset of specific adverse events [9]. Hence, there is a heavy reliance on RWD for post-authorisation safety studies to monitor adverse effects of medical and pharmacological interventions for cancer management.

A series of studies in PCBaSe have investigated adverse effects following hormonal treatment for PCa. In 2010, Van Hemelrijck et al. [55] assessed the risk of cardiovascular events and deaths following PCa diagnosis compared to risk in the Swedish population and found highest risk among men who received androgen deprivation therapy (ADT). O’Farrell et al. extended this work to study the timing of cardiovascular disease (CVD) risk associated with common types of ADT [56]. Men with a recent history of CVD had a particularly high risk of an additional CVD event shortly after starting on gonadotrophin-releasing hormone agonists (GnRH). The authors also noted that estimates of increased CVD risk from RCTs were not as high as those from studies using RWD [56]. While biases due to selection of men for different types of ADT may explain differences in risk estimates, RWD better reflect prescribing practices among clinicians and, hence, provide a truer picture of risks in the broader community. The findings from these studies, along with other observational studies [57,58,59,60], led to the FDA issuing a requirement for manufactures of GnRH to add safety warnings about the risk of CVD [61].

Subsequent studies have also reported higher risk of diabetes [62], fractures [63], thromboembolic events [64,65] and non-Alzheimer’s dementia [66] among men on GnRH. Comparing men on GnRH agonists with those on anti-androgens, Beckmann et al. found that the overall comorbidity burden was substantially higher following GnRH treatment [67]. Collectively, these studies support the notion that ADT, particularly GnRH agonists, should only be used when there is a strict indication, with monitoring of men with a prior recent CVD event to mitigate subsequent risks. Furthermore, they support the practice of initially prescribing anti-androgens for advanced, non-metastatic PCa to reduce ADT-associated morbidity, a practice which is not common outside of Scandinavia.

### 4.4. Treatment Patterns/Disparities in Access to Care

It is only possible to determine treatment patterns and disparities in care utilising RWD. Such studies are particularly important in the context of PCa given the variety of treatment options and the rapid emergence of novel treatments for very advanced disease. Population-based registries and representative cohorts allow assessment of “real-world practice” regarding dissemination of novel therapies, disparities in access and compliance with treatment guidelines.

Since its inception, the NPCR has been utilised to describe patterns of care in routine general practice and to identify disparities in the provision of optimal care. For example, Berglund et al. (2011) showed that radical treatment for high-risk PCa differed significantly according to socioeconomic status (i.e., class of occupation), which likely also contributed to differences in PCa mortality. In a subsequent study, Tomic et al. showed that men with higher incomes were more likely receive curative treatment and had shorter waiting times for prostatectomy [68]. These studies point to socioeconomic disparities in PCa management, despite universal healthcare across Sweden.

Several studies have also identified disparities in treatments according to age. Bratt et al. (2015) noted undertreatment of high-risk PCa among men aged 70–79 years, with life expectancy >10 years and little or no comorbidity. Orrason et al. [69] showed a marked increase in radical treatment, mainly radiotherapy, for advanced localised PCa (cT3/4, M0/X), including among older men, though there were still large disparities across regions.

While clinical registers generally collect data on primary treatments, allowing for good descriptions of primary care patterns, data on subsequent treatments are generally more limited. Linkage with PDR in PCBaSe provides data on ADT as a secondary treatment. For example, Lycken et al. [70] investigated the use of ADT among 45,000 men diagnosed with localised PCa (1997–2009) who underwent curative or deferred treatment. Twelve percent of these men were eventually treated with ADT, with a large variation in start of ADT (4 years for watchful waiting to 17 years for RP in low-risk cases). Other important studies examining patterns of care in advanced disease include the use of novel ARTs (i.e., enzalutamide and abiraterone) [71], chemotherapy [72] and palliative medications (e.g., glucocorticoids, antidepressants, anxiolytic and opioids) before death [73]. Each of these revealed disparities in prescribing patterns according to age, education level and region of residence, highlighting the need for more equitable access.

**Figure 3:**
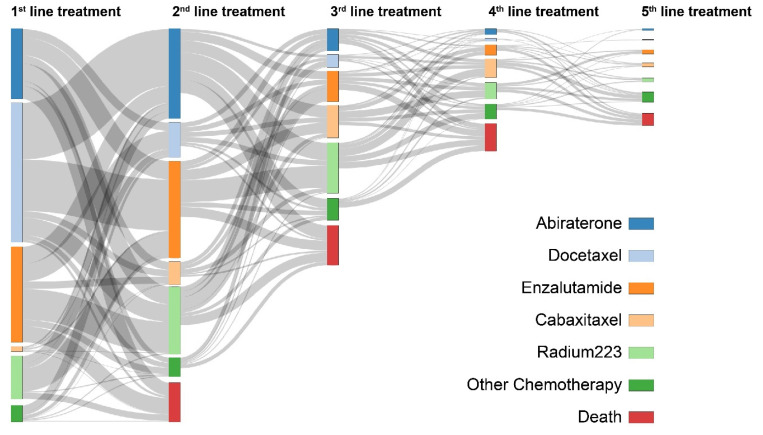
Sankey diagram showing the sequence of systemic therapies among men who developed castrate-resistant prostate cancer (CRPC) by October 2019 (*n* = 3400) from their initial presentation of castrate resistance.

As noted previously, shortcomings due to the lack of follow-up data in the NPCR were addressed through prospective follow-up of men with advanced PCa from participating centres within the PPC. The utility of PPC data to describe treatment complexity in very advanced PCa is illustrated in Figure 3, showing the sequence of systemic therapies for approximately 3400 men who had reached castrate resistance by October 2019. Commonly, first-line treatment for CRPC involved chemotherapy with docetaxel or an androgen receptor-targeted drug (ART), e.g., enzalutamide or abiraterone.

In 2018, Swedish authorities extended approval for subsidised use of abiraterone to include de novo metastatic hormone-sensitive prostate cancer (mHSPC), with some additional caveats, based on favourable trial results and EMA approval. Fallara et al. [74] recently assessed the uptake of abiraterone for this extended indication using the Swedish Board of Health and Welfare’s “Statistics Pathway”, which provides rapid data linkage between clinical registers and recent prescription data in PDR (updated monthly). Their results showed a gradual increase in the use of abiraterone for mHSPC, from 4% in the second quarter of 2018 to 26% in the third quarter of 2020. Again, considerable variation was observed across the 21 regions in Sweden, with use ranging from 0% to 39%.

### 4.5. Health Economics

The recent study by Svensson demonstrated the utility of PPC for health economics research [75]. In this study, researchers examined the time spent in various phases of advanced disease and associated healthcare costs. The high price of ARTs was identified as the main factor contributing to higher total cost of care during castrate-resistant phase [75]. Using PCBaSe’s linkage with the PDR, a subsequent study assessed whether time on ARTs for CRPC was excessive, based on dates of prescription among men who started on either abiraterone or enzalutamide. Duration on ARTs was found to be shorter in clinical practice (4 months for abiraterone and 8 months for enzalutamide) than in the RCTs (14 and 17 months, respectively). [76].

### 4.6. Guideline Compliance/Quality Indicators/Impact on Policy and Practice

Another important role of RWD is quality assurance to drive improvements in cancer care. Quality assurance activities utilising RWD from NPCR include assessment of guideline compliance and their impact on practice [77,78,79], reporting of quality indicators [80,81] and rapid feedback on performance to healthcare providers [82]. For example, Robertson et al. [81] assessed waiting times from referral to initiation of curative treatment for breast, colorectal and prostate cancer from several clinical cancer registers, including the NPCR. Men with PCa had by far the longest waiting times, varying from 117 to 280 days across regions, emphasising the need for improved care pathways to facilitate more rapid diagnosis and treatment. Makarov et al. [79] studied the impact of regular feedback to clinicians to reduce inappropriate imaging to stage PCa. Over a 10-year period, inappropriate use of bone scanning in men with low-risk PCa decreased from 45% in 1998 to 3% in 2009 [79].

Several other examples demonstrate the value of RWD to influence policy and practice. Swedish guidelines have recommended AS for men with low-risk PCa since 2007. However, an NPCR study by Loeb et al. [78] indicated lower-than-optimal rates of AS update across Sweden. Base on further work using NPCR data [83], revised guidelines now recommend that all men with very low-risk disease (i.e., low risk plus additional features: PSA density < 0.15 ng/mL/cc, ≤4 positive cores and <8 mm total cancer in cores) be managed by AS [84]. Adherence to this recommendation has increased, with 91% of men with very low-risk PCa initially undergoing AS [85].

**Figure 4:**
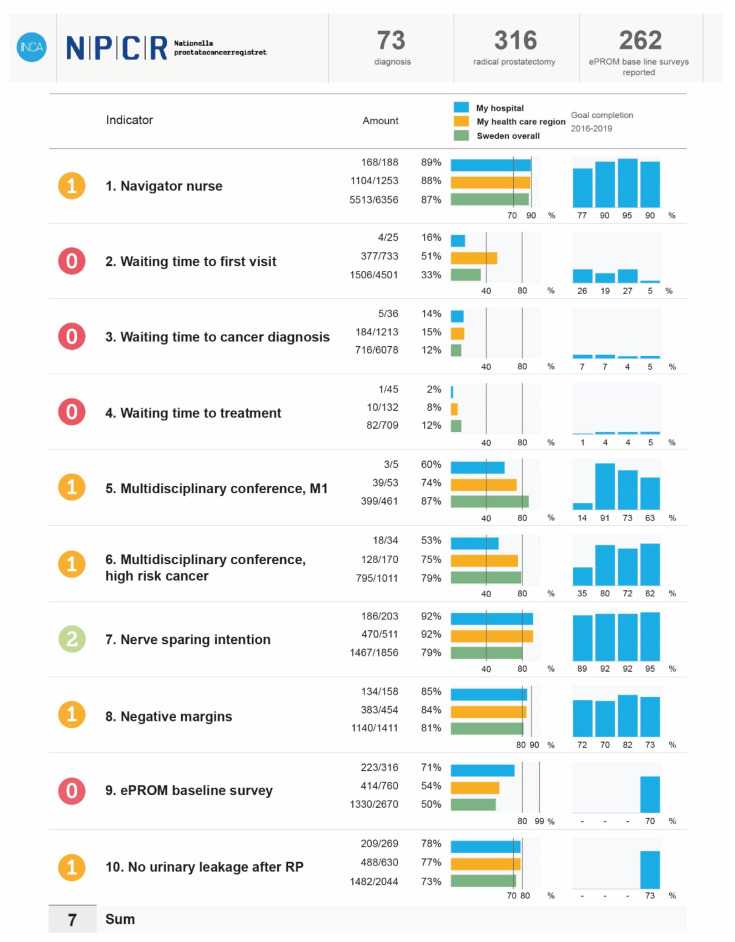
Quality indicator “Dashboard” for urologists. Definition of indicators (per treatment centre/county council/region) for new diagnoses during the reporting period: 1. Proportion of men with newly diagnosed prostate cancer who have a named contact nurse; 2. Proportion of men seen within a specialist clinic for suspected prostate cancer within 14 days of initial referral; 3. Proportion of men receiving notification of cancer diagnosis within 11 days of their prostate biopsy; 4. Proportion of men with a time interval between referral and initiation of curative primary treatment less than 61 days (radical prostatectomy, RP), 58 days (radiotherapy (RT) without neoadjuvant hormone therapy) or 50 days (neoadjuvant hormone therapy with RT) for men with high-risk cancer; 5. Proportion of men up to 80 years of age with primarily metastatic disease (M1), whose case was discussed at a multidisciplinary conference (MDC). The definition of a multidisciplinary conference in the National Prostate Cancer Register (NPCR) is that both a urologist and an oncologist participate; 6. Proportion of men up to 80 years with high-risk cancer without distant metastases, whose case was discussed at MDC. High-risk cancer is divided into localized high-risk cancer or locally advanced cancer; 7. Proportion of people who underwent surgery with a low- or intermediate-risk tumour who underwent intra/interfascial nerve-sparing resection; 8. Proportion of prostatectomy specimens with negative resection margins at pT2.; 9. Proportion of men who underwent prostatectomy who completed a baseline patient-reported outcome survey; 10. Non-severe urinary incontinence 12 months after radical prostatectomy.

Quality improvement can also be achieved through direct feedback of performance to care providers. In 2014, the NPCR created the “What’s going on, Prostate Cancer” dashboard to provide timely, at-a-glance feedback on performance measures for PCa care for individual practice units [82]. Ten readily actionable quality indicators were selected for inclusion in the dashboard based on the indicators and performance targets established in the 2014 Swedish National Prostate Cancer Guidelines [84]. These covered both administrative and procedural aspects of PCa care such as waiting times, designated clinical nurse specialist, multidisciplinary team review, appropriate diagnostic work up, compliance with specific treatment guidelines and quality outcomes for certain treatment (e.g., clear surgical margins). Using continually updated data for patients seen within their practice, the dashboard visually displays a three-tiered scale for each performance indicator, with intermediate cut-offs below those set in the guidelines to incentivise quality improvement [82]. Figure 4 shows an example of the “Dashboard” display used for urologists. In addition, the NPCR has created a public online interactive reporting system (available at www.npcr.se/RATTEN, accessed on: 20 December 2020) [86]. This reporting system, available in Swedish and in English, allows the viewer to query the database regarding pattern of care down to hospital level. Data are updated biannually.

## 5. Strengths and Limitations

Strengths of NCPR/PCBaSe include the nation-wide population coverage, inclusion of PCa-free “controls” and linkages to other national databases, which provides a broad scope for addressing a range of research questions. However, limitations exist with respect to the type of data available in NPCR and PCBaSe.

The lack of data on the frequency and results of PSA testing is a major limitation in the NPCR. The lack of follow-up PSA data limits the assessment of important clinical outcomes such as biochemical recurrence and castrate resistance. Collection of PSA data in population-wide registries, including the NPCR, is hampered by multiple laboratories providing PSA assessment and lack of a centralized repository. Several regional centres have accessed PSA and biopsy data—for example, the Stockholm PSA and Prostate Biopsy Register [87]—and inclusion of such data in PCBaSe nationally is being planned.

Access to population-wide data on lifestyle and social factors which influence, or are impacted by, PCa is limited. Several NPCR studies have included lifestyle factors though linkage to survey data obtained through workplace health assessments [41] and from attendees at charity events [42]. However, the potential for bias in such studies is high due to the selective nature of workplace cohorts or “volunteer” survey participants. While some data on social factors are available through linkage with LISA (employment status, sick leave, income, marital status, etc.), utilization of these data has been limited to date.

Lack of follow-up in NPCR/PCBaSe has also been problematic, with limited data on secondary treatments, functional outcomes, quality-of-life and clinical endpoints other than mortality. As for most population-based registries, limited resources have prevented longitudinal follow-up of the ever-increasing number of registered PCa cases (now >200,000 in the NPCR). Additional collection of detailed data on all prostatectomies and radical radiotherapy within the NPCR, including secondary treatments, has addressed this limitation to some extent. Furthermore, PPC was primarily established for longitudinal follow-up of subsequent treatments and clinical, functional and quality-of-life outcomes in men with metastases. At present, treating clinicians/centres voluntary participate in the PPC, though there are ongoing efforts toward reaching population-wide coverage in the future. Consideration of future “electronic” real-time capturing of data may provide another solution to longitudinal follow-up.

A further limitation, which applies equally to population-based registries in other jurisdictions, is the potential lack of generalizability of findings beyond Sweden. The Swedish population is quite homogeneous (i.e., predominantly Caucasian), and some policies and practices vary from other countries. Nonetheless, many of the study findings from NPCR/PCBaSe will be applicable to other regions with similar populations profiles/clinical guidelines or provide hypotheses that can be tested elsewhere.

## 6. Impact on Knowledge and Practice

The use of RWD from PCBaSe/NPCR has influenced clinical practice in Sweden (and beyond) in numerous ways. For example, data on familial risk of intermediate/high-risk PCa are utilised in counselling men on PSA testing and treatment decisions [36]. Studies have indicated that there is no need for additional monitoring for PCa risk in kidney transplant recipients receiving immunosuppressive therapies [40] or men on testosterone therapy [29]. Outcome studies have supported the use of AS for low-risk PCa [45] and, conversely, curative treatment for locally advanced prostate cancer [46]. Subsequently, increasing use of radiotherapy has been observed [47] and an RCT has commenced assessing radiotherapy versus RP [48] in men with locally advanced PCa. There has been a ten-fold decrease in the use of unnecessary bone scans in men with low-risk PCa, in line with the “Choose Wisely” campaign which utilized NPCR data [79]. Studies examining the side effects of ADT, along with other real-world evidence, have led to the FDA recommending labelling of GnRH packaging, warning of increased CVD risk [55,61]. Recent economic studies have supported Swedish authorities’ decision to approve the use of novel ADTs for metastatic-CRPC from a cost perspective [76], given that time on these medications is not excessive. Disparities in PCa care and non-compliance with guidelines have also been described, leading to revised guidelines with stronger recommendations and subsequent improvements in clinical practice relating to AS for low-risk PCa [85] and radical treatment for advanced PCa [69]. The development of the performance indicator “dashboard” for clinicians has also driven quality improvements in PCa care [86].

Similar examples are seen in other registry data sources around the world [12].

## 7. Future Directions

One of the exciting directions being planned for the NPCR is the development of a platform for conducting registry-based randomised clinical trials (R-RCTs). R-RCTs are a subgroup of what are termed “pragmatic trials”, a methodology that aims to bridge the gap between tightly controlled experimental study designs and observational study designs utilising RWD [88]. R-RCTs can be thought of as “prospective randomized trials that use a clinical registry for one or several major functions for trial conduct and outcomes reporting” [89]. As such, they rely on RWD sources. The contribution of registers can be through a variety of means including identifying patients, randomisation, collecting data on baseline and treatment characteristics, assisting with recruitment and consent and identifying and adjudicating on clinical endpoints.

R-RCTs combine the advantages of RCTs and observational “real-world” studies [90]. Since the aim of R-RCTs is to test the effectiveness of interventions in routine practice rather than their efficacy under ideal conditions, by design, they are generally less restrictive and less concerned with achieving high rates of compliance. They tend to have high external validity due to their larger sample sizes, more heterogeneous study populations and diverse settings. However, inclusion of a randomisation component serves to strengthen internal validity as well. In addition, R-RCTs are substantially less expensive and resource-intensive than classical RCTs [90].

While not a substitute for conventional RCTs to establish efficacy prior to approval, R-RCTs are an ideal methodology for evaluating existing treatment options which are already available and widely used in clinical practice. This is very relevant to PCa, given the numerous treatments options available. As a quality clinical register with population-wide coverage and extensive clinical detail, the NPCR is well positioned to facilitate R-RCTs. The future development of a randomisation function within the NPCR will be modelled on the successful R-RCT initiative in the Swedish cardiology register, Swedish Web System for Enhancement and Development of Evidence-based Care in Heart Disease Evaluated According to Recommended Therapy (SWEDEHEART) [91,92]. R-RCTs in SWEDEHEART have been highly influential in changing cardiology practice. For example, findings from the Thrombus Aspiration in ST-Elevation (TASTE) trial led to recommendations that thrombus aspiration not be performed for myocardial infarction and the subsequent decline in practice [93].

One limiting factor for establishing R-RCTs in the NPCR is the delay in registration, resulting in many men having received primary treatment before being enrolled in the NPCR. Addressing this will require parallel development of real-time data collection within the NPCR, which is also currently underway.

## 8. Conclusions

Much of our knowledge of PCa has been gained through RWD. This will continue to be the case with the growth and improvement of clinical cancer registries, increased opportunities through data linkage with administrative data sources and requirement for evidence that reflects broader community practice. RWD complement evidence from clinical trials, both in terms of generating hypotheses that can be tested in RCTs and validating/extending trial findings to examine effectiveness outside of controlled settings to include long-term outcomes, adverse effects and delivery of care. Additionally, RWD are an important source of evidence when RCTs are not feasible or will never be undertaken, i.e., for understanding aetiology, evaluating interventions that are well established in clinical practice and assessment of rare adverse events. As exemplified by data in the NPCR, PPC and PCBaSe, clinical registries in Sweden will continue to be of great value as a source of RWD to improve outcomes for men affected by PCa.
